# Paraoxonase 2 Induces a Phenotypic Switch in Macrophage Polarization Favoring an M2 Anti-Inflammatory State

**DOI:** 10.1155/2015/915243

**Published:** 2015-12-08

**Authors:** Marie Koren-Gluzer, Mira Rosenblat, Tony Hayek

**Affiliations:** ^1^The Lipid Research Laboratory, Technion Faculty of Medicine, The Rappaport Family Institute for Research in the Medical Sciences, and Rambam Health Care Campus, 31096 Haifa, Israel; ^2^Internal Medicine E Department, Rambam Health Care Campus, 31096 Haifa, Israel

## Abstract

Inflammatory processes are involved in atherosclerosis development. Macrophages play a major role in the early atherogenesis, and they are present in the atherosclerotic lesion in two phenotypes: proinflammatory (M1) or anti-inflammatory (M2). Paraoxonase 2 (PON2) is expressed in macrophages, and it was shown to protect against atherosclerosis. Thus, the aim of our study was to analyze the direct effect of PON2 on macrophage inflammatory phenotypes. Ex vivo studies were performed with murine peritoneal macrophages (MPM) harvested from control C57BL/6 and PON2-deficient (PON2KO) mice. PON2KO MPM showed an enhanced proinflammatory phenotype compared to the control, both in the basal state and following M1 activation by IFN*γ* and lipopolysaccharide (LPS). In parallel, PON2KO MPM also showed reduced anti-inflammatory responses in the basal state and also following M2 activation by IL-4. Moreover, the PON2-null MPM demonstrated enhanced phagocytosis and reactive oxygen species (ROS) production in the basal state and following M1 activation. The direct effect of PON2 was shown by transfecting human PON2 (hPON2) into PON2KO MPM. PON2 transfection attenuated the macrophages' response to M1 activation and enhanced M2 response. These PON2 effects were associated with attenuation of macrophages' abilities to phagocyte and to generate ROS. We conclude that PON2 promotes an M1 to M2 switch in macrophage phenotypes.

## 1. Introduction

Inflammatory processes are involved in atherosclerosis development [1]. Macrophages play a major role in the early atherogenesis [2, 3], and they are present in the atherosclerotic lesion in two phenotypes: proinflammatory (M1) or anti-inflammatory (M2) [4–7]. In the plaque, serum lipids, serum lipoproteins, and various pro- or anti-inflammatory stimuli such as cytokines, chemokines, and small bioactive molecules could greatly influence the macrophage phenotype inducing switch towards more proinflammatory or anti-inflammatory properties. The M1/M2 balance in plaques is dynamic, with M1 predominating in disease progression and M2 in regression [8–11]. In vitro, the classic macrophage activation M1 is caused by the cytokine IFN*γ* in combination with lipopolysaccharide (LPS), whereas the alternative macrophage activation (M2) is caused by the cytokines IL-4 and IL-13 [12–14]. Recently, it was shown both in vitro and in vivo that pomegranate polyphenols directly suppress macrophage inflammatory responses and promote macrophage phenotype switch from M1 to M2 [15]. Understanding the mechanisms of macrophage plasticity and resolving functional characteristics of distinct macrophage phenotypes should help in the development of new strategies for treatment of chronic inflammation in atherosclerosis [16, 17]. Paraoxonase 2 (PON2) is an intracellular enzyme that is widely expressed in almost every tissue including macrophages [18, 19]. Several studies indicate a major role for PON2 in attenuation of atherosclerosis development [20–23]. PON2's antiatherogenic properties include protection of arterial wall cells from oxidative stress and apoptosis [18, 19, 24–26] and also from triglyceride accumulation [27]. PON2 is expressed also in immune cells, and it hydrolyzes 3OC (12)-HSL, a quorum-sensing molecule produced by gram-negative microbial pathogens [28, 29]. PON2 plays an important role in hepatic insulin signalling and underscores the influence of macrophage-mediated inflammatory response on hepatic insulin sensitivity [30]. The mechanisms contributing to the generation of proinflammatory or anti-inflammatory macrophage phenotype during atherosclerosis development are not fully understood. Paraoxonase 1 (PON1), another member of the paraoxonase gene family that protects against atherosclerosis development [31], is not expressed in macrophages [18], and it is present in the circulation associated with HDL. A recent study, using peritoneal macrophages or bone marrow-derived macrophages from PON1 transgenic mice which express human PON1, an artificial nonphysiological status, demonstrated that PON1 reduces the inflammatory response to M1 stimulation [32]. Since PON2 possesses different antiatherogenic properties than PON1 and since PON2 is normally expressed in human and mouse macrophages, the aim of the present study was to assess the direct effect of PON2 on the polarization of macrophages. For this purpose we used MPM from PON2KO mice in comparison to control C57BL/6 MPM. In addition, we transfected human PON2 into the PON2KO MPM. The effect of PON2 on both M1 and M2 activation was analyzed.

## 2. Materials and Methods

### 2.1. Chemicals

Phosphate buffered saline (PBS), Dulbecco's modified Eagle's medium (DMEM), fetal calf serum (FCS) (heat-inactivated at 56°C for 30 min), penicillin, streptomycin, L-glutamine, and sodium pyruvate were from Biological Industries (Beth Haemek, Israel). 2′,7′-Dichlorodihydrofluorescein diacetate (DCFH-DA) and lipopolysaccharide (LPS) from* Salmonella typhimurium* were from Sigma Aldrich (St. Louis, MO, USA). Recombinant murine interferon-gamma (IFN*γ*) and interleukin-4 (IL-4) were from PeproTech (Rocky Hill, NJ, USA).

### 2.2. Animals

Five-week-old male C57BL/6 mice were purchased from Jackson Laboratories (Bar Harbor, ME). The PON2-deficient mice on the C57BL/6 background were generated as previously described [20] and were a generous gift from Dr. Srinivasa T. Reddy, Atherosclerosis Research Unit, Division of Cardiology, Department of Medicine at UCLA, Los Angeles, CA, USA. We used only male mice in our study. The mice were bred under pathogen-free conditions in the animal facility of the Faculty of Medicine (Technion-Israel Institute of Technology, Haifa, Israel). The research was conducted in conformity with the Public Health Service Policy on Human Care and Use of Laboratory Animals and approved by the Committee for Supervision of Animal Experiments, the Technion-Israel Institute of Technology, Haifa, Israel.

### 2.3. Mouse Peritoneal Macrophages (MPM)

MPM were prepared from mice that were sacrificed by overanesthesia, and MPM were harvested prior to removal of the aorta, from the peritoneal fluid, 3 days after intraperitoneal injection into each mouse of 3 mL of thioglycolate (24 g/L) in saline. The cells (10–20 × 10^6^/mouse) were washed and centrifuged three times with phosphate buffered saline (PBS) at 1000 g for 10 min and then resuspended to 10^9^/L in DMEM containing 15% horse serum (heat-inactivated at 56°C for 30 min), 0.1 U/L penicillin, 100 mg/L streptomycin, and 2 mmol/L-glutamine. The cell suspension was dispensed into 35 mm plastic Petri dishes and incubated in a humidified incubator (5% CO_2_, 95% air) for 2 h. The dishes were washed once with 5 mL DMEM to remove nonadherent cells, and the monolayer was then incubated under similar conditions for 18 h, prior to the beginning of the experiment.

### 2.4. Proinflammatory (M1) and Anti-Inflammatory (M2) Activation

Cells were activated with either LPS (100 ng/mL) and IFN*γ* (20 ng/mL) or IL-4 (20 ng/mL) for increased periods of times up to 30 hours. Cytokine secretion reached a maximal level after 16 hours. Thus, incubation for 16 hours was employed in all experiments for measuring cytokine secretion and mRNA expression.

### 2.5. Cytokine Secretion

The levels of cell-released TNF*α*, IL-6, and IL-10 were measured in the collected incubation medium and determined by using DuoSet ELISA development systems (R&D Systems, Inc., Minneapolis, MN, USA) following manufacturer's instructions. All reactions were run at room temperature. Optical density was determined and analyzed by the KC4 microplate reader (BIO-TEK, Instruments Inc., Winooski, VT, USA).

### 2.6. Cytokines, Arginase I, and Arginase II mRNA Expression

RNA was extracted from cells using MasterPure RNA purification kit (Epicentre Biotechnologies, Madison, WI, USA). cDNA was prepared using Verso cDNA kit (Thermo Scientific, Epsom, UK). Primers and probes for genes were designed by Primer Design, Southampton, UK, using ABsolute Blue QPCR ROX mix (Thermo Scientific), and expression was determined by quantitative real-time PCR with a Rotor-Gene 6000 amplification detection system following manufacturer's instructions.

### 2.7. Assessment of Phagocytosis

Phagocytosis tests were performed using 2 *μ*m fluorescent blue-green latex beads (Sigma Aldrich, St. Louis, MO, USA). Latex beads were opsonized by incubation in 1 : 1 ratio of latex bead and mice serum for 2 hours at 37°C. Opsonized latex beads were then suspended in 1 mL DMEM supplemented with 5% FCS and added to macrophages for 4 h at 37°C. Macrophages were washed twice with PBS to remove nonphagocytosed material, scraped, and then analyzed for the uptake of FITC-coupled beads by FACS.

### 2.8. Detection of Reactive Oxygen Species (ROS) Production

ROS production was assayed through the oxidation of DCFH-DA. MPM cells were seeded on 12-well plate in DMEM supplemented with 5% FCS and incubated at 37°C/5% CO_2_ until reaching 50% confluency. M1 activation cells were washed with PBS, suspended in 100 *μ*L of PBS, and incubated with 10 *μ*mol/L DCFH-DA for 30 min at 37°C. Reaction was stopped by washing the cells with PBS. Seven measurements of cellular fluorescence determined by FACS were done at 510 to 540 nm, after excitation of the cells at 488 nm with an argon ion laser. Ten thousand events were registered for each experiment. Cellular fluorescence was measured using FACS analysis with a FACS Calibur flow Cytometer (Becton-Dickenson, San Jose, CA), and data were analyzed using CellQuest software (BD Biosciences, San Jose, CA, USA).

### 2.9. Transfection of MPM with Human PON2

MPM from PON2-deficient mice were transfected with 2 *μ*g/mL of plasmid DNA (human PON2 (hPON2) gene in pcDNA3.1+ plasmid or with the empty pcDNA3.1+ plasmid, a generous gift from Dr. Dragomir Draganov, University of Michigan, Ann Arbor, MI) in Dulbecco's modified Eagle's medium containing 3 *μ*L/mL of FuGene 6 reagent (Roche).

### 2.10. Statistical Analysis

Each experiment was performed in triplicate, and each individual experiment was replicated three times (*n* = 3) in order to achieve statistical significance meaning. Statistical analyses used Student's *t*-test for comparing differences between two groups and one-way ANOVA, followed by the Student-Newman-Keuls test, for comparing differences between multiple groups. Results are given as mean ± SD.

## 3. Results

### 3.1. MPM from PON2-Deficient Mice (PON2KO) Display Enhanced Proinflammatory Activation

**Figure 1:**
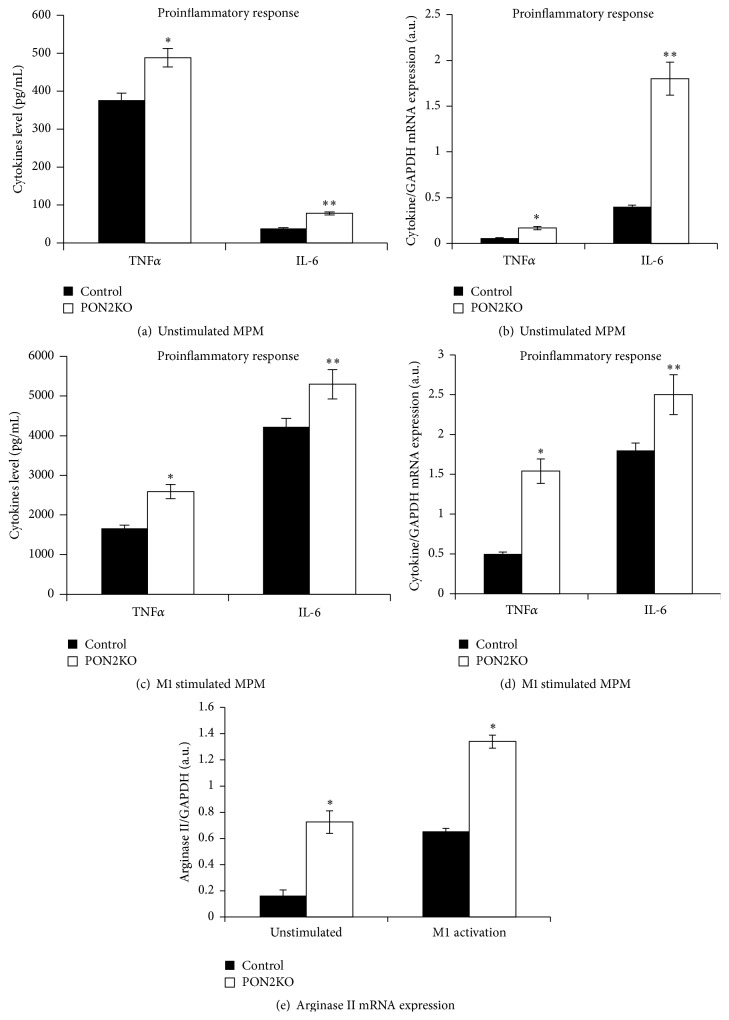
MPM from PON2KO display increased proinflammatory activation. MPM were harvested from 3-month-old control (C57BL/6) male mice and from age matched PON2KO mice. The cells were stimulated toward M1 activation with LPS (100 ng/mL) and IFN*γ* (20 ng/mL) for 12 hours. Secretion of TNF*α* and IL-6 to the medium was measured under basal (a) or M1 stimulation (c) conditions. TNF*α* and IL-6 mRNA expression, analyzed by quantitative PCR and normalized to GAPDH, were measured in the cells under basal (b) or M1 stimulation (d) conditions. Arginase II mRNA expression was also measured under basal or M1 stimulation conditions (e). Results are expressed as mean ± SD (*n* = 3), ^*∗*^ *P* < 0.03, PON2KO versus control MPM.

In MPM harvested from PON2KO mice, spontaneous secretion of TNF*α* and IL-6 (Figure 1(a)) and basal mRNA expression (Figure 1(b)) of TNF*α* and IL-6 were significantly elevated by 1.3-, 2-, 2.8-, and 4.5-fold, respectively, in comparison to MPM from C57BL/6 control mice. M1 activation induced by IFN*γ* and LPS stimulated TNF*α* and IL-6 secretion (Figure 1(c)) and expression (Figure 1(d)) in comparison to unstimulated cells (Figures 1(a) and 1(b)) in MPM from both mice groups. However, both TNF*α* and IL-6 secretion (Figure 1(c)) and mRNA (Figure 1(d)) expression in response to M1 activation were significantly increased by 1.5- and 1.3-fold and by 3- and 1.4-fold, respectively, in PON2KO MPM in comparison to control MPM.

In addition, arginase ΙΙ mRNA expression (expressed by M1 macrophages) was 4.5-fold higher in unstimulated PON2KO MPM cells as compared to MPM obtained from control mice (Figure 1(e)). M1 activation of MPM from both mice groups stimulated arginase ΙΙ mRNA expression in comparison to unstimulated cells. However, in M1-activated PON2KO MPM, arginase ΙΙ mRNA expression increased 2.2-fold as compared to control MPM. Collectively, these results suggest that PON2 expression in macrophages is associated with protection against proinflammatory stimuli.

### 3.2. MPM from PON2KO Display Reduced Anti-Inflammatory Activation

**Figure 2:**
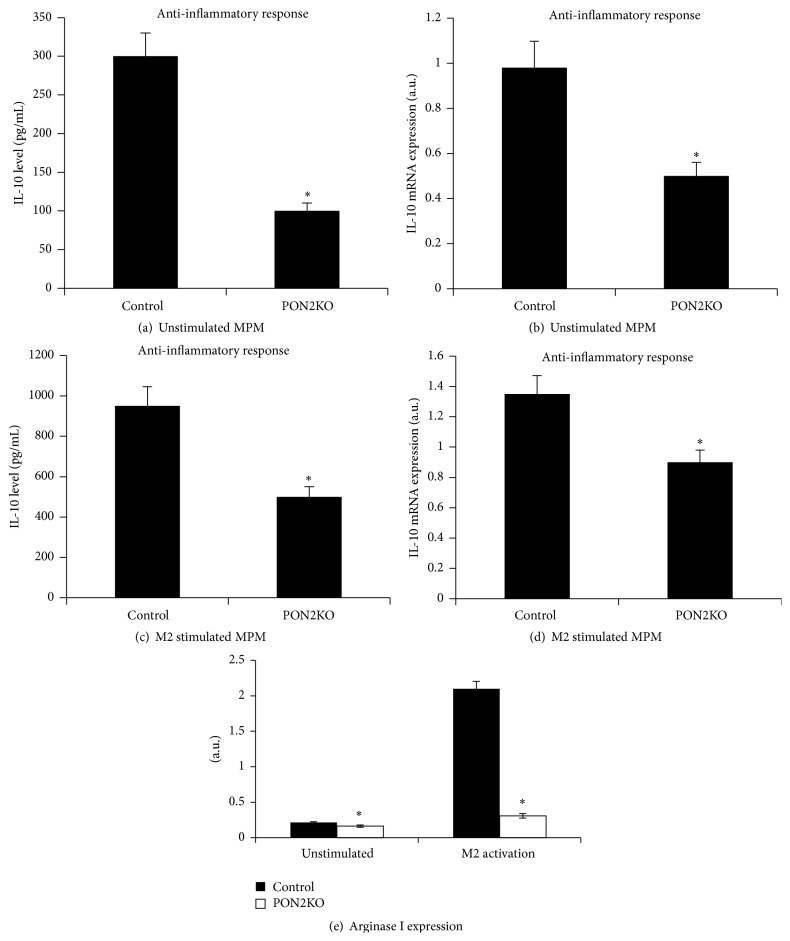
MPM from PON2KO display reduced anti-inflammatory activation. MPM were harvested from 3-month-old control (C57BL/6) male mice and from age matched PON2KO mice. The cells were stimulated toward M2 activation with IL-4 (20 ng/mL) for 12 hours. Secretion of IL-10 to the medium was measured under basal (a) or M2 stimulation (c) conditions. IL-10 mRNA expression, analyzed by quantitative PCR and normalized to GAPDH, was measured in the cells under basal (b) or M2 stimulation (d) conditions. Arginase I mRNA expression was also measured under basal or M2 stimulating conditions (e). Results are expressed as mean ± SD (*n* = 3), ^*∗*^ *P* < 0.01, PON2KO MPM versus control MPM.

Next, MPM harvested from control and PON2KO mice were subjected to M2 activation induced by IL-4. Spontaneous IL-10 secretion (Figure 2(a)) and basal mRNA expression (Figure 2(b)) were significantly reduced by 67% and 50%, respectively, in PON2KO MPM in comparison to control MPM. M2 activation stimulated IL-10 secretion (Figure 2(c)) and mRNA expression (Figure 2(d)) in comparison to unstimulated cells in MPM from both mice groups (Figures 2(a) and 2(b)). However, in response to M2 activation, both IL-10 secretion (Figure 2(c)) and mRNA expression (Figure 2(d)) were significantly lower in PON2KO MPM versus control MPM by 49% and 33%, respectively.

In parallel, the basal arginase Ι mRNA expression (Figure 2(e)) in PON2KO MPM was significantly lower 1.5-fold as compared to control MPM. After M2 stimulation it was 3-fold lower in PON2KO MPM compared to the level of expression in control MPM. Taken altogether, these results indicate that PON2 expression by macrophages not only inhibits macrophage response to classical M1 activation, but also promotes macrophage polarization toward the M2 alternative phenotype.

### 3.3. PON2 Modulates Macrophage M1 Functional Phenotype

**Figure 3:**
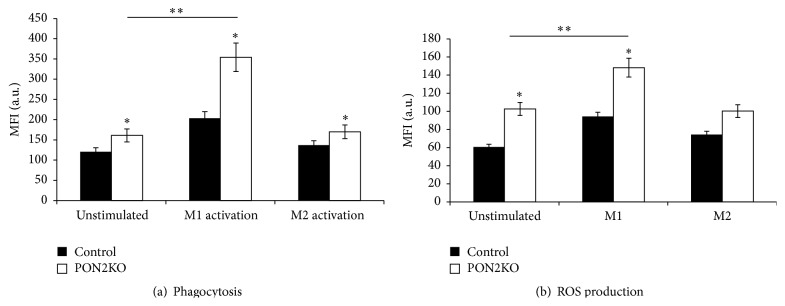
M1 functional phenotype in PON2KO MPM versus control mice MPM. MPM from control or PON2KO mice were either nontreated or activated to the M1 or M2 phenotype. (a) Phagocytosis was determined in cells that were incubated with FITC-conjugated latex beads for 4 hours and analyzed using FACS. Results are given as mean fluorescence intensity (MFI). (b) ROS production was analyzed by FACS and is expressed as MFI. Results are expressed as mean ± SD (*n* = 3), ^*∗*^ *P* < 0.05, PON2KO MPM versus control MPM; ^*∗∗*^ *P* < 0.01, M1 stimulated versus unstimulated cells.

Next, we determined whether PON2 affects macrophage functions that are operative in M1, including ROS production and phagocytosis. We measured the phagocytosis of FITC-labeled latex beads by M1- or M2-activated MPM from control and PON2KO mice.  Figure 3(a) demonstrates an increment in latex particle phagocytosis in PON2KO MPM compared to control MPM in unstimulated cells as well as M1-activated macrophages by 1.3- and 1.7-fold, respectively. Following M2 activation, macrophage phagocytosis ability was also enhanced in PON2KO MPM versus control MPM by 1.24-fold and was similar to unstimulated cells (Figure 3(a)). In parallel, the level of ROS production (Figure 3(b)) from resting unstimulated macrophages was 1.7-fold elevated in PON2KO MPM compared to control MPM. M1 activation induced a remarkable increase in ROS production in comparison to resting unstimulated macrophages. ROS production in M1 stimulated MPM increased 1.6-fold in PON2KO MPM as compared to control MPM (Figure 3(b)). M2 activation did not affect MPM ROS production as compared to unstimulated cells. These results suggest that macrophage PON2 inhibits M1-induced ROS formation and phagocytosis.

### 3.4. Human PON2 (hPON2) Promotes Macrophage Polarization toward an Anti-Inflammatory M2 Phenotype

**Figure 4:**
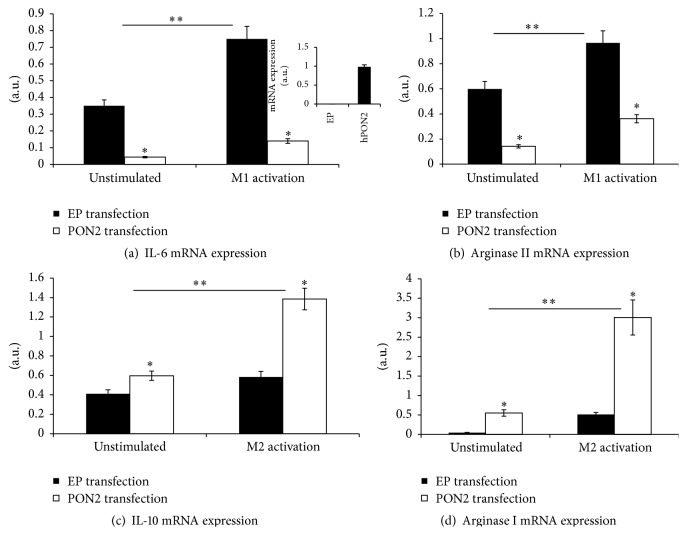
Direct effect of human PON2 (hPON2) on the inflammatory and anti-inflammatory responses in PON2KO MPM. MPM were harvested from 3-month-old PON2KO male mice. The cells were transfected with pcDNA3.1+ empty plasmid (EP) or with pcDNA3.1+ plasmid containing hPON2 for 4 hours at 37°C. Cells were then washed and cultured with DMEM medium + 10% fetal calf serum for 48 hours. The cells were cultured for additional 12 hours unstimulated and then either M1-activated with IFN*γ*/LPS or M2-activated with IL-4. IL-6 (a), arginase ΙΙ (b), IL-10 (c), and arginase Ι (d) mRNA expressions were determined by quantitative PCR and results were normalized to GAPDH expression. The inset of (a) shows hPON2 mRNA expression in PON2-deficient MPM transfected with EP and PON2KO MPM transfected with hPON2. Results are expressed as mean ± SD (*n* = 3), ^*∗*^ *P* < 0.01, PON2KO MPM versus control MPM; M1 stimulated versus unstimulated, ^*∗∗*^ *P* < 0.001, M1, M2 stimulated versus unstimulated cells.

To further confirm the direct inhibitory effect of PON2 on macrophage inflammatory response, we reintroduced PON2 to PON2KO MPM by transfecting them with a vector containing the hPON2 plasmid or with an empty plasmid (EP) as a control. The expression of hPON2 in the transfected cells was confirmed by quantitative PCR (inset of Figure 4(a)). After transfection (48 hours), the cells were untreated or activated toward M1 with LPS + IFN*γ* or toward M2 with IL-4, and we measured M1 and M2 markers accordingly. Compared to EP, transfection of hPON2 to PON2KO MPM significantly inhibited MPM inflammatory responses as reflected by a decrease in IL-6 (Figure 4(a)) and in arginase ΙΙ mRNA expression (Figure 4(b)), in unstimulated cells by 89% and by 75%, respectively, and after M1 activation by 80% and 65%, respectively. In parallel, compared to EP, hPON2 transfection to PON2KO MPM showed an enhanced MPM anti-inflammatory response reflected by increased IL-10 (Figure 4(c)) and arginase Ι mRNA expression (Figure 4(d)) in unstimulated cells 1.5- and 11-fold, respectively, and in M2 activation 2.8- and 6-fold, respectively.

These results indicate that indeed PON2 not only inhibits macrophage response to classical M1 activation, but also promotes macrophage polarization toward the M2 anti-inflammatory phenotype.

### 3.5. hPON2 Modulates Macrophage M1 Functional Phenotype

**Figure 5:**
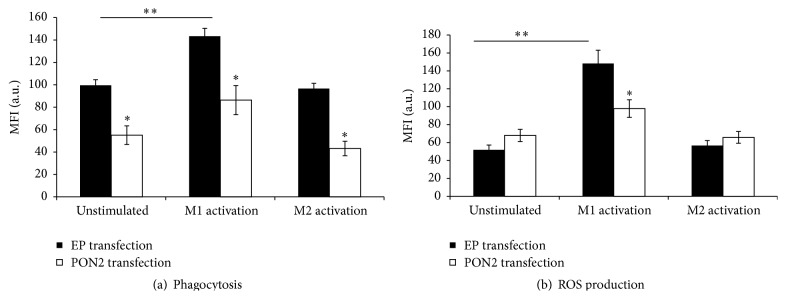
Direct effect of hPON2 on M1 functional phenotype in PON2KO MPM. MPM obtained from 3-month-old PON2KO male mice were transfected with pcDNA3.1+ empty plasmid (EP) or with pcDNA3.1+ plasmid containing hPON2 for 4 hours at 37°C. Cells were then washed and cultured with DMEM medium + 10% fetal calf serum for 48 hours. The cells were cultured for an additional 12 hours either unstimulated or stimulated with IFN*γ*/LPS or IL-4. (a) Phagocytosis was determined in cells that were incubated with FITC-conjugated latex beads for an additional 4 hours and analyzed using FACS. Results are given as mean fluorescence intensity (MFI). (b) ROS production was measured after 45 minutes of incubation with DCFH by FACS and is expressed as MFI. Results are expressed as mean ± SD (*n* = 3), ^*∗*^ *P* < 0.01, PON2KO MPM versus control MPM; ^*∗∗*^ *P* < 0.01, M1 stimulated versus unstimulated cells.

Compared to EP, transfection of hPON2 to PON2KO MPM inhibited macrophage phagocytosis in unstimulated cells, as well as M1-activated cells and M2-activated cells by 43%, 38%, and 54%, respectively (Figure 5(a)). PON2 transfection to PON2KO MPM had no statistically significant effect on the macrophages' ability to generate ROS, as compared to MPM transfected with EP. However, in M1 stimulated cells, macrophage ROS production was significantly attenuated upon hPON2 transfection by 32% as compared to cells transfected with an EP (Figure 5(b)). No statistically significant effect of hPON2 transfection on MPM ROS production was noted under M2 activation versus unstimulated cells or versus cells with EP transfection.

## 4. Discussion

In the present study we demonstrate that macrophage PON2 directly attenuated the proinflammatory phenotype, in both unstimulated and M1 stimulated peritoneal macrophages. In addition, PON2 induced a phenotypic switch in macrophage polarization favoring an M2 anti-inflammatory phenotype.

We used in our study peritoneal macrophages from PON2KO mice in comparison to MPM from control C57BL/6 mice, which express PON2. M1 classic activation was induced by IFN*γ* + LPS following determination of TNF*α*, IL-6, and arginase II expression. All these parameters were significantly higher in the PON2KO versus control MPM. The M2 alternative activation was induced by IL-4 following determination of IL-10 and arginase I expression. All these parameters were significantly lower in the PON2KO versus control MPM. Functionally, PON2 deficiency was associated with increased ROS formation, in both unstimulated and M1 stimulated MPM in accordance with previous studies [19, 24, 25], and with enhanced latex particles phagocytosis.

Direct role of macrophage PON2 in the macrophage inflammatory responses was assessed by transfecting human PON2 into PON2KO MPM. This procedure resulted in a significant decrement in TNF*α*, IL-6, and arginase II expression and in parallel a significant increment in IL-10 and arginase I expression. The reverse of these responses by transfecting PON2KO macrophages with the human PON2 gene clearly indicates that PON2 plays an important role in polarization of macrophages from M1 toward the M2 phenotype. It could be that PON2-induced reduction in macrophage oxidative stress leads to the observed changes in the expression of the proinflammatory versus anti-inflammatory cytokines.

In a previous study it was shown that PON1 reduces macrophage inflammatory response [32]. PON1 unlike PON2 is not expressed in macrophages [18], and unlike PON2 it is present in the circulation associated with HDL. In that study [32] they used macrophages (MPM of BMDM) from human PON1 transgenic mice. These macrophages express human PON1, but this is artificial state, not physiological one. In addition the authors used recombinant PON1 incubation with J774A.1 macrophages, but there is no free PON1. PON1 can contribute to HDL anti-inflammatory activity. In that study [32] the authors did not measure arginase I and II expression or used M2 activation like we did. The novelty of our study is the use PON2KO MPM and PON2KO MPM transfected with human PON2.

PON1 and PON2 were both shown to protect from atherosclerosis development [31], secondary to HDL-associated PON1 antiatherogenic effects in the circulation and in the arterial wall and to PON2 antiatherogenic effects only in the arterial wall. Both enzymes were shown to reduce oxidative stress [24], but PON2 protects also from triglyceride accumulation [27], whereas PON1 protects from cholesterol accumulation, by inhibiting macrophage cholesterol biosynthesis [33] and by stimulating of HDL-mediated cholesterol efflux [34]. In both PON2KO MPM and C57BL/6 MPM, cellular cholesterol metabolism was similar, as noted by similar macrophage cholesterol mass and similar rates of cholesterol biosynthesis and of HDL-mediated cholesterol efflux [35]. In PON2KO MPM versus C57BL/6 MPM, there was a significant increase in the cellular triglyceride content and in the rate of macrophage triglyceride biosynthesis [35].

PON1 was also shown to protect from diabetes development in mice [36], and PON2 was shown to affect hepatic insulin signaling [31] and protected macrophages from high glucose-induced oxidative stress and triglyceride accumulation [37].

The progression of the atherosclerotic plaque in terms of size and nature is mediated by the inflammatory status of the macrophages in the vascular wall. Advanced atherosclerotic lesions are characterized by activated macrophages dominated by the M1 subset in all stages [4, 8] whereas in early lesions macrophages express markers corresponding to the M2 phenotype [9]. In addition, M2 macrophages were suggested to be involved in inflammation remission [11].

In summary, our results support the role of PON2 in protecting against atherosclerosis development by shifting the polarization of macrophages toward the M2 anti-inflammatory phenotype. Understanding the mechanisms of macrophage plasticity and resolving functional characteristics of distinct macrophage phenotypes should help in the development of new strategies for treatment of chronic inflammation in atherosclerosis.
